# A Bayesian Modeling Approach to Examine the Role of Testosterone Administration on the Endowment Effect and Risk-Taking

**DOI:** 10.3389/fnins.2022.858168

**Published:** 2022-07-20

**Authors:** Mikhail Votinov, Irina Knyazeva, Ute Habel, Kerstin Konrad, Andrei A. Puiu

**Affiliations:** ^1^Research Center Jülich, Institute of Neuroscience and Medicine, JARA-Institute Brain Structure Function Relationship (INM 10), Julich, Germany; ^2^Department of Psychiatry, Psychotherapy and Psychosomatics, Faculty of Medicine, RWTH Aachen University, Aachen, Germany; ^3^N.P. Bechtereva Institute of Human Brain, Russian Academy of Science, Saint Petersburg, Russia; ^4^Saint Petersburg State University, Saint Petersburg, Russia; ^5^Department of Child and Adolescent Psychiatry, Psychosomatics and Psychotherapy, Faculty of Medicine, RWTH Aachen University, Aachen, Germany

**Keywords:** testosterone, decision-making, risk-taking, Bayesian analysis and modeling, prospect theory, endowment effect, sex hormones

## Abstract

Financial risk-taking and loss aversion are multifaceted phenomena that are the focus of neuroscience, psychology, and economics research. A growing number of studies highlighted the role of hormones (particularly of testosterone) on socio-economic decision-making. However, the effects of testosterone on risk-taking under framing and consumer-based choices and preferences are inconclusive. We investigated the effects of 100 mg testosterone administration on aspects of decision-making within the Prospect Theory framework which is the most used descriptive model of decision-making under risk. We assessed risk-taking under framing and the endowment effect (effect of possession) using Bayesian modeling. Forty men participated in this double-blind placebo-controlled fully-randomized cross-over experiment and performed two tasks. One was a risk-taking task with binary choices under positive and negative framing associated with different probabilities. In the second task participants had to bid money for hedonic and utilitarian items. We observed a significant increase in serum testosterone concentrations after transdermal application. Compared to placebo, testosterone administration increased risk-taking under the positive framing (very large effect size) and decreased under the negative framing (moderate to small). The sensitivity to gain was positive in each framing. Our model showed that decision-making is jointly influenced by testosterone and the trade-off between gains and losses. However, while the endowment effect was more pronounced for hedonic than for utilitarian items, the effect was independent of testosterone. The findings provide novel information on the complex modulatory role of testosterone on risk-taking within the framework of prospect theory and shed light on mechanisms of behavioral economic biases. The proposed models of effects of individual differences in testosterone on risk-taking could be used as predictive models for reference-depended behavior under positive and negative framing with low and high probabilities.

## Introduction

In everyday decision-making, we weigh the risks and rewards (i.e., probability) associated with competing outcomes in terms of relative payoffs and the potential for negative outcomes (i.e., gains vs. losses). For decades scientists have been investigating decisions under risk and ambiguity and proposed several models that quantify decision-making.

One of the most influential frameworks for researching decision-making under risk is the prospect theory (PT; Kahneman and Tversky, 1979; Tversky and Kahneman, 1991). The theory posits that decisions are affected by how potential outcomes (prospects) are cognitively represented in terms of gains, losses, and their associated probabilities. As such, rational individuals aim to optimize outcomes by weighing the value, probability, and cumulative wealth. According to PT, the value *V*(*x, p*) of a simple prospect that pays *x* with probability p and can be represented as:


(1) $$\begin{array}{l} V\left(x,p\right)=v\left(x\right)\omega \left(p\right) \end{array}$$

where *v*(*p*) measures the subjective value of the consequence *x*, and measures the impact of probability *p* on the attractiveness of the prospect (Tversky and Kahneman, 1991; Stanton, 2017).

According to prospect theory, the value function *V*(*x, p*) exhibits the psychophysics of diminishing sensitivity with the value function being steeper for losses (loss aversion) than for gains. In other words, a loss of €50 is felt more than a gain of €50. Loss aversion reflects a tendency to avoid losses relative to acquiring equivalent gains. The utility of monetary payoffs that depends on previous experiences is what distinguishes loss from risk aversion. Overall, prospect theory argues that decision-makers weigh gains and losses differently, weighing perceived gains more than perceived losses. This shows that humans think in terms of expected utility relative to a reference point (i.e., current financial situation/wealth) rather than in absolute outcomes.

A refinement of the original version of prospect theory, cumulative prospect theory (CPT) applies different weights to the cumulative probability distribution function instead of applying weights to the probabilities of individual outcomes (Tversky and Kahneman, 1992). This ensures that the value of an outcome is multiplied by a decision weight and not by an additive probability (for a model overview, please see Fox and Poldrack, 2009). A recent study across 19 countries and over 4,000 participants replicated the empirical foundations of the prospect theory and showed that none of the prospect theory's theoretical constructs (i.e., isolation effect, framing effects, overweighting of small probabilities, etc.) from the original study were unreliable (Ruggeri et al., 2020). These findings confirm that prospect theory is a robust descriptive model of decision-making under risk and uncertainty.

Unlike risky decision-making, riskless choice relates to prospect theory through the so-called endowment effect (EE) (Thaler, 1980; Kahneman et al., 1991). The endowment effect has been used as evidence for theories of reference-dependent preferences and loss aversion and it shows that the minimum amount of money people are willing to accept (WTA) when selling an item is significantly higher than the minimum amount they are willing to pay (WTP) for the same item if they do not already own it (Kahneman et al., 1991; Ariely et al., 2005; Votinov et al., 2010, 2013; Camerer, 2011; Gächter et al., 2021). Endowing a person with a good (even hypothetically), seems to establish a reference point people move away from reluctantly or if they are paid a large sum (Camerer, 2011). This observation underscores a status quo bias toward own goods. Furthermore, an extreme feeling of possession may lead to an excessive acquisition of items resulting in experiencing difficulties discarding them, which can be linked to hoarding disorder (Pushkarskaya et al., 2020). In another words the EE is a byproduct of loss aversion in a way that the losing things is more painful than enjoyment of gaining them.

The type of goods (utilitarian vs. hedonic) that consumers buy or sell heavily influences the WTA/WTP ratio. Utilitarian goods focus on function, practicality, and are generally more of what people “need” as opposed to hedonic goods which provide more fun, are not as functional, and reflect what people “want” (Hirschman and Holbrook, 1982; Strahilevitz and Myers, 1998). Buying and selling hedonic instead of utilitarian goods has previously been found to increase the WTA/WTP ratio (Chan, 2015). For example, one field survey (Dhar and Wertenbroch, 2000) demonstrated this by collecting data from two hundred seventeen MBA students about the features of the car they owned. Participants rated their car on a scale from one to nine for hedonic dimensions and then separately for utilitarian dimensions. They were then asked how much money they would be willing to sell their car for. As predicted, those with a more hedonic car would demand more money concerning their car's market price than those with more utilitarian cars would. Another study by Cramer and Antonides (2011) tested decision making with respect to hedonic vs. utilitarian food products. They observed strong endowment effect for hedonic compared to utilitarian food products and proposed that status quo bias for hedonic food products may lead to relatively unhealthy food choices. A potential reason for this disparity may be because the owners of hedonic goods may develop a more significant symbolic relationship with those goods than with utilitarian goods (Belk, 1988).

Testosterone is one of the major steroid hormones produced in men and women (though in different quantities) that modulates brain activity by binding to intracellular androgen receptors and regulating gene expression via genomic and non-genomic effects (for a comprehensive review, see Brinkmann, 2011). Developments in neuroeconomics and neuroendocrinology highlighted shared biological mechanisms that support decision-making involving economic risk-taking. As such, testosterone is an interesting biomarker for socioeconomic decision-making and for status seeking behaviors.

Engaging in risky behaviors might be an evolutionary strategy to attain higher positions in social hierarchies where status is important. It is therefore likely that testosterone also modulates risk-taking behaviors and several studies reliably associated testosterone with social and economic decision-making under high risk (Eisenegger et al., 2011; Apicella et al., 2014). A recent meta-analysis on studies measuring endogenous levels of testosterone showed a significant albeit small correlation between testosterone and risk-taking which was not influenced by gender nor by outcome measures (Kurath and Mata, 2018). Nevertheless, several other studies reported contrasting see Mehta et al. (2015) or null results (Van der Loos et al., 2013; Derntl et al., 2014; Nadler et al., 2021; Stanton et al., 2021). However, it should be noted that these studies used different approaches with some measuring basal testosterone in saliva (Derntl et al., 2014; Mehta et al., 2015), some in the serum (Van der Loos et al., 2013), while the rest (Nadler et al., 2021; Stanton et al., 2021) administered testosterone exogenously.

Steroid hormones including testosterone preferentially modulate brain activity (Dreher et al., 2007; Votinov et al., 2020) regulating the neural function of areas involved in economic decision-making and emotion regulation. Several studies measured endogenous testosterone levels and found that high testosterone concentrations correlate well with increased risk-taking in both men and women thus indicating a heightened willingness to take financial risks (Garbarino et al., 2011; Stanton et al., 2011a; Chicaiza-Becerra and Garcia-Molina, 2017). Moreover, some studies showed a positive relationship between basal endogenous testosterone levels and decreased risk-aversion thus showing that testosterone may circumstantially promote greater risk neutrality (Apicella et al., 2008; Sapienza et al., 2009; Stanton et al., 2011a). Other studies investigated the effects of exogenous (pharmacologically-elevated) testosterone on risky decision-making. In men, transdermal testosterone shifted investments toward riskier assets (Cueva et al., 2015) and increased risk-taking under conditions of unknown probabilities during strategic decision-making (Goudriaan et al., 2010; Wagels et al., 2017). Exogenous testosterone increased risk-taking in the Iowa Gambling Task (IGT) in a group of women, likely by increasing reward sensitivity while decreasing punishment sensitivity. Similarly, endogenous testosterone accounted for 11% decision-making in the uncertainty phase of the IGT (Singh, 2021).

The literature on testosterone and risk-taking is far from conclusive. For instance, several studies (Zethraeus et al., 2009; Boksem et al., 2013) found no effect of exogenous testosterone (sublingual administration) on women's risk attitudes or ambiguity tolerance. Likewise, in a double-blind within-subjects study investigating risk-taking and loss aversion, Stanton and colleagues (2021) found no consistent relationship between pharmacologically-elevated testosterone and economic decisions (Stanton et al., 2021). Instead, they argue that findings may be explained by several situational moderators.

Not only is testosterone associated with risk-taking and decision-making but it also influences consumer behavior by altering consumer-based choices and preferences. For instance, prenatal testosterone exposure correlates well with men's courtship-related gift-giving (Nepomuceno et al., 2016a,b) in an attempt to attain a certain status. The study investigated the influence of testosterone on men's preferences for positional goods and found that administering testosterone increases men's preference for status brands compared to brands of similar perceived quality but lower perceived status. Testosterone further increases positive attitudes toward positional goods when they are described as status-enhancing (Nave et al., 2018). Consumer choices are thus directly linked to competition and competing interests (Durante and Griskevicius, 2016) which are also subject to endocrine effects (Cherki et al., 2021).

While several studies investigated how people may experience the endowment effect and what role testosterone plays in some aspects of loss aversion, the interaction between testosterone and endowment effect has not yet been thoroughly explored. Similarly, the effects of pharmacologically-elevated testosterone on context-depended risk taking-taking have not been investigated.

As such, we aim to investigate the role of testosterone on risk-taking and loss aversion under positive and negative framing.

First, we examine whether testosterone administration affects decision-making in a series of gambles under positive and negative framing (gain and loss) associated with various probabilities. Under testosterone administration, we expect participants to show increased risk-seeking behaviors regardless of framing type.

Second, we investigate testosterone's effect on loss aversion (endowment effect) during the economic valuation of two goods categories (utilitarian vs hedonic). We hypothesize that the endowment effect (and therefore the WTA/WTP ratio) will be higher for hedonic compared to utilitarian goods. Compared to placebo, we expect testosterone administration to further increase the endowment effect (and the WTA/WTP ratio) for both types of goods.

## Methods

### Socio-Demographics Data

Forty healthy male participants with mean age 23.2 ± 2.9, participated in the study. These individuals concurrently took part in a larger, separate functional MRI study and agreed to take part in this study additionally. Due to technical problems hormonal data was recorded for 39 participants (missing data was replaced by mean values for each group). For the Endowment task, we analyzed data from 37 subjects because two participants did not complete the task while another participant's data was removed because a value of zero was indicated when selling/buying items on more than 30% of the items. The study was approved by the local Ethics Committee of the Medical Faculty and were in accordance with the Helsinki declaration (1964) and its later amendments or comparable ethical standards. All participants gave informed consent prior to participation and received financial compensation for their participation in the study.

### Testosterone Application

Participants performed the tasks twice separated by a washout period of a minimum of 7 days. They arrived at the laboratory between 8.30 a.m. and 8.45 a.m. and were briefed about the procedure and signed the consent form. A first 10 mL blood sample (baseline) was collected around 9 a.m. After collecting the first blood sample, the transdermal gel was immediately applied by the same research assistant to participants' scapular area and let to dry out for 15 min. Participants were then kept under observation and were instructed not to engage in physical activities. The second blood sample was taken 4.15 h after the treatment administration. The testosterone gel consisted of Testotop® (active testosterone ingredient), 96% ethanol, polyacrylate and propylene glycol, disodium EDTA, trometamol, and purified water. On the second visit, they received a placebo gel containing the same ingredients as above but excluding the active testosterone ingredient (Testotop®). Treatment order was counterbalanced and randomized and the protocol was previously validated (Puiu et al., 2019). Half of the participants received the testosterone gel first and placebo second. Participants performed the task 1.5 h following testosterone/placebo administration so as to allow for testosterone's loading period (Puiu et al., 2019). The design of the study was double-blind, meaning that neither researchers nor participants were aware what treatment was administered when. At the end of each session, we asked the participants and experimenter to state what treatment they thought was administered.

#### Hormone Profiles

Testosterone and cortisol serum concentrations were analyzed by electrochemiluminescence immunoassays (ECLIA, Roche®Diagnostics GmbH). For testosterone, the inter-assay coefficient was 2.4 with a lower detection limit of 0.09 nmol/L. Respective inter-assay data for cortisol was 3.8 with a lower detection limit of 0.054 μg/dl. Intra-assay coefficients for testosterone and cortisol were below 3. All analyzes were conducted under strict internal and external quality control at the Clinical Chemistry, Haematology, Virology, and Microbiology Laboratory Diagnostic Centre (LDZ) of the RWTH Aachen University Hospital.

### Experiment Design

**Figure 1:**
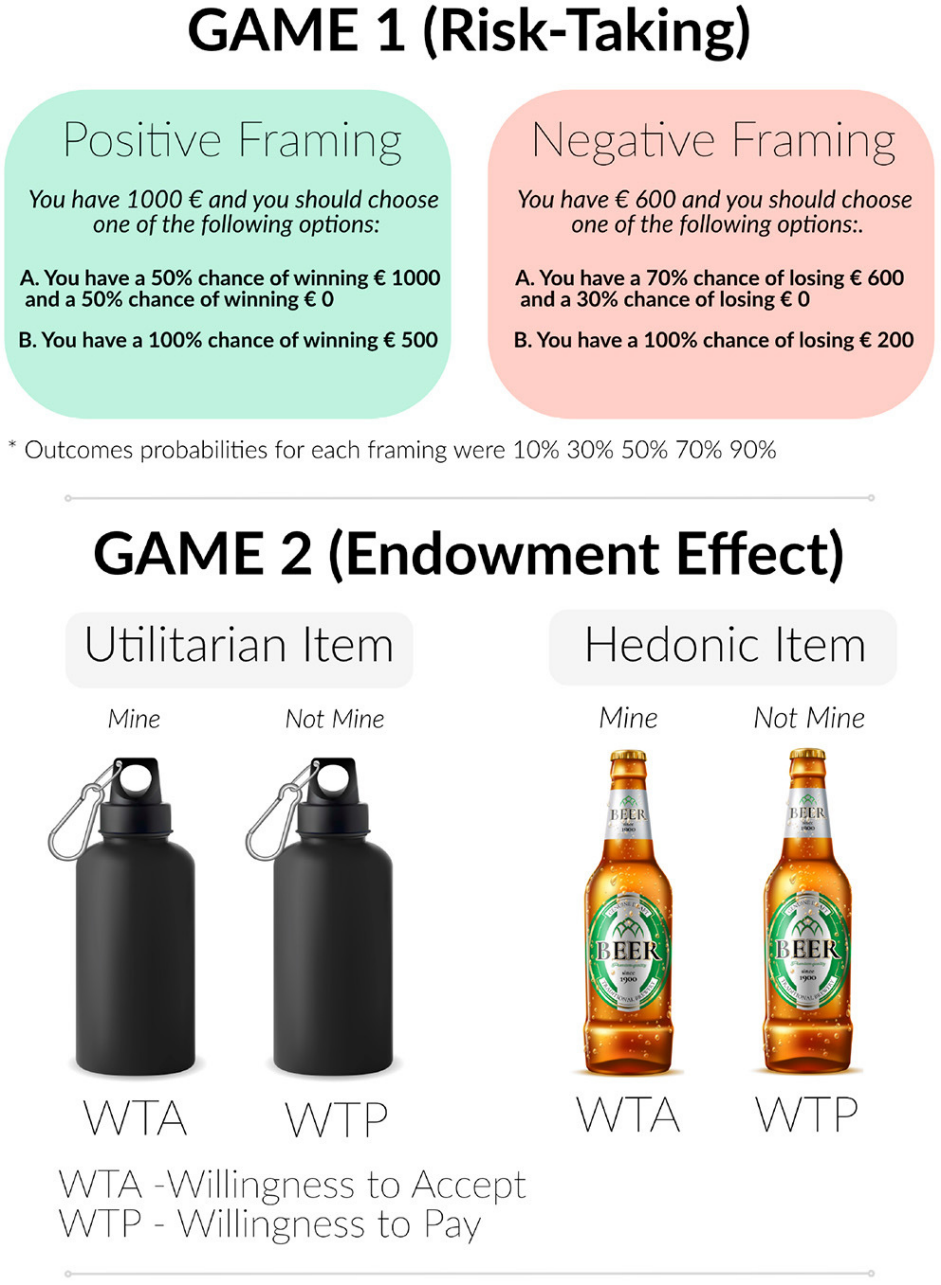
Experimental design of 2 games.

The experimental setup consisted of a risk-taking and an endowment task see Figure 1.

#### Risk-Taking Task

Participants made a choice between two options where one was a sure option and another one a gamble option with different probabilities (*p*) of winning and losing. There were five binary choices under two framings. Under positive framing, option A reflects winning a larger amount of money or getting nothing. Option B indexes sure winning but with a lesser payoff (i.e., amount of money). Under negative framing, option A reflects losing more money or not losing anything. Option B reflects a sure loss of a small amount (Figure 1, Game 1 exemplifies some questions and the options for the framing type). Each option was associated with the following probability sets: 0.1, 0.3, 0.5, 0.7, and 0.9 for both framings (see Table 1 for the probability distributions for all options). All games were presented in a pseudorandom order and participants played the game twice (once under placebo and once under testosterone administration).

**Table 1:** Game types.

	**Probabilities**	**Positive**		**Negative**	
	**P (risky)**	**A (risky win)**	**B (sure win)**	**A (risky loss)**	**B (sure loss)**
Game 5	0.9	2,000	1,500	2,400	1,200
Game 4	0.7	500	250	600	200
Game 3	0.5	1,000	500	1,000	500
Game 2	0.3	500	250	1,800	600
Game 1	0.1	250	50	1,200	400

#### Endowment Effect Task

For this task, participants were presented with two categories of goods:

(1) hedonic goods, which can be described as items that one desires because they are attractive and bringing fun
(2) utilitarian goods, which are items that are rather practical and goal-oriented.

Photographs of all items were taken from the Amazon online shop. All items were pretested in a preliminary behavioral experiment on a separate group of participants to see if it is easy to distinguish between two categories. Each item was presented twice randomly during each session (placebo and testosterone), and participants had to estimate the items' value (in Euro). When it was indicated that they own an item, participants were asked to indicate the minimum amount of money they were willing to accept (WTA) to give it up. When the item belonged to someone else, participants were asked to indicate the maximum amount of money they were willing to pay (WTP) to acquire it.

## Data Analysis

We used Python (v3.8) for data preprocessing and analyzes. Preprocessing and exploratory data analyzes were performed with pandas (v1.2) and seaborn (v 0.11) libraries. Bayesian statistical analysis and modeling were performed with a custom code based on PyMC3 (v 3.11) (Python package for Bayesian statistical modeling and probabilistic machine learning) and openly accessible through Jupyter notebooks containing detailed research procedures, methods, and results. A detailed description of every step for each analysis with the code and data are available at GitHub and OSF. Bayesian modeling involves making choices concerning prior distributions, likelihood functions and model evaluation, which includes posterior checks and quality assessments (Aczel et al., 2020). Here we provided and briefly described the models we used for data analysis. Full model specification and the description of estimation procedures are available in the Supplementary File.

### Risk-Taking Under Framing

The generally accepted model of people making decisions under risk is based on the cumulative prospect theory (Tversky and Kahneman, 1992). According to prospect theory (Fox and Poldrack, 2009), making a choice between two gambling options is based on a subjective value that represents its desirability to the decision maker. The subjective value of money for option O *V*(*O*), where option O is payoff *x* with probability *p* is given by Equation (1). The subjective value of payoff is defined as a power function from payoff:


(2) $$v(x_{i})=\{\begin{array}{ll} x^{\alpha }, & \text{if}(x)>0\\ -\lambda {\left(-x\right)}^{\alpha }, & \text{else} \end{array}$$

This form of value function exhibits the psychophysics of diminishing sensitivity to the value of payoff. The weighting function captures the diminishing sensitivity to changes in probability. Diminishing sensitivity implies an inverse s-shape weighting function that is concave near zero and convex near one. This shape reflects the observed risk attitudes with underweighted high probabilities and overweighted low probabilities. Here we used a single parameter function as suggested in Tversky and Kahneman (1992).


(3) $$\begin{array}{l} \omega \left(p\right)=\frac{p^{c}}{{\left[p^{c}+{\left(1-p\right)}^{c}\right]}^{1/c}} \end{array}$$

According to the prospect theory, a rational decision maker should always choose an option with a larger subjective value. To include possible errors caused by noise, cognitive states or other factors, an additional sensitivity parameter ϕ is introduced to the choice model. A 0–1 choice probability is usually described by a sigmoid transformation. Finally, the choice probability transforms in the following way:


(4) $$\begin{array}{l} p\left(A,B\right)=\frac{e^{\phi V\left(A\right)}}{e^{\phi V\left(A\right)}+e^{\phi V\left(B\right)}}=\frac{1}{1+e^{-\phi \left(V\left(A\right)-V\left(B\right)\right)}} \end{array}$$

In case of equal options, we have a random choice with a 0.5 probability. When option A has a bigger subjective value, the expression e^−ϕ(*V*(*A*)−*V*(*B*))^ tends to zero and the probability to choose option A tends to 1. According to the previous studies, the typical value for α lies in ranges [0.75, 0.95] and for *c*- [0.6, 0.96] (Fox and Poldrack, 2009).

#### Modification of CPT Model to Hormone Dependent Risk-Taking Framing Paradigm

To model risk-taking and choice behavior in our framing paradigm, we used a Bayesian approach for several reasons. First, Bayesian modeling allows us to estimate prior knowledge about decision-making processes. Second, the output of a Bayesian model is an interpretable parameter with clear posterior distributions that may be used as an alternative to *p*-values. Last, there are several studies where hierarchical Bayesian approach for CPT was successfully applied (Shiffrin et al., 2008; Nilsson et al., 2011, 2020).

The choice rule (see Equation 4) may be rewritten as a general linear model with the logit link function (inverse to logistic function) (McElreath, 2018):


logit(p)=ϕ*gap(A,B,α,c),

where α, *c*- value function and choice probability correction from CPT theory parameters and gap(*A, B*, α, *c*) = *V*(*A*, α, *c*)−*V*(*B*, α, *c*).

We introduced an intercept to the choice rule because, in practice, considering equal subjective value, a sure option is more often preferred over a risky one. The modification with the intercept can be mathematically expressed as follows:


(5) $$\begin{array}{l} \text{logit}\left(p\right)=\text{Intercept}+\text{Beta}*\text{gap}\left(A,B,\alpha ,c\right) \end{array}$$

In previous studies (Fox and Poldrack, 2009) the differences between the options (gap) ranged very little (up to 10 $). Our experimental setup entailed range changes between 250 and 1,200 Euro. To reduce scale sensitivity, we used log1*p* transformation for the gap:


logGap=sign(gap)log(1+abs(gap))

With this transformation, small gap values remain approximately the same because log(1 + *x*) ~ *x* and big values are diminished. We suggest that both framing options (F) have their own Intercept and Beta parameters. The final model for choice rule was estimated using


(6) $$\begin{array}{l} \text{logit}\left(p\right)={\text{Intercept}}_{F}+{\text{Beta}}_{F}*\text{logGap}\left(A_{F},B_{F},\alpha ,c\right), \end{array}$$

where Intercept and Beta depend on the serum testosterone level in the following way:


(7) $$\{\begin{array}{l} \text{Intercept}=a_{F}+\delta \_a_{F}*\text{Tchange},\\ \text{Beta}=b_{F}+\delta \_b_{F} \end{array}$$

where Tchange indicates the change in testosterone level. Participants took part in both framings and each hormone condition, so Intercept (shift to sure option) may correlate with the slope Beta (sensitivity to the gap). Moreover, since both sets of the parameters are connected to risk attitudes, we estimated a hierarchical model with covariance between all parameters. More information is provided in Supplementary.

#### Prospect Theory Parameters Re-estimation

In the current experiment, we had five measurements for each individual in each framing. The primary aim of this study is not to explore prospect theory parameters but to examine the testosterone application effect on risk-taking in different framing. To simplify, we assumed that CPT parameters have no dependence on hormonal application. Thus, we estimated CPT parameters α, *c*, based on data from previous decision-making studies. The closest experimental setup to ours that had an open dataset available was described and analyzed in Nilsson et al. (2011). We replicated the hierarchical model suggested in the original paper but only for pure gain and pure loss (positive and negative framing) games, as we did not have mixed games in our study. After that we estimated the model with log transform modification and used the estimated parameters α and *c* in our choice model. The results of the replication study with and without log transform modification are available in the Supplementary Materials and accompanying code.

### Endowment Effect Exploration

We used standard hypothesis testing and Bayesian modeling to investigate the endowment effect. The following hypotheses were tested:

1. **Endowment effect regardless of the type of goods**
  Formally:
  - H0: *WTA*/*WTP* = 1 against
  - H1: *WTA*/*WTP* > 1
  A one-tailed *t*-test was performed to check this hypothesis. In addition, for each item we modeled a *WTA*/*WTP* ratio using multilevel Bayesian regression (Nilsson et al., 2011; McElreath, 2018). There are two cluster variables in this experiment: item and participant. In other words, each observation has an item and a participant indicator. To model this, we constructed a group-level hyperparameter describing the overall average WTA/WTP ratio (μ_0_) with two varying intercept parameters responsible for variations through goods *g*_item_ and subjects μ_ind_. Since the WTA/WTP value is strongly positive, we used a logarithmic link function, so that the parameters μ_0_, μ_ind_, *g*_item_ covered the entire real line:
$$\log\left({\text{WTA/WTP}}_{\left[\text{ind,item}\right]}\right)=\mu_{0}+\mu_{\text{ind}}+g_{\text{item}}$$
  As a result, we were able to estimate the endowment ratio for each item as a posterior distribution for exp(μ_0_ + *g*_item_). Full model specification with the quality assessment is provided in Supplementary Material and in source code.
2. **Differences between hedonic and utilitarian items**. Afterwards, we tested the differences between hedonic and utilitarian goods to examine whether hedonic items had significantly higher WTA/WTP ratio than utilitarian items.
  - H0: WTA/WTP_*h*_ = WTA/WTP_*u*_
  - H1: WTA/WTP_*h*_ > WTA/WTP_*u*_
  This hypothesis was tested with a one-sided *t*-test. We also estimated the posterior distribution for the hedonic and utilitarian WTA/WTP ratio using type of goods as a cluster variable, accounted for by the following model:
$$\log\left({\text{WTA/WTP}}_{type}\right)=\mu_{type}.$$
3. **Differences between testosterone and placebo** We used repeated-measures analyzes of variance (ANOVA) to examine the differences between testosterone and placebo groups and modeled individual item effects using a Bayesian model. We used the same model as the one described above and introduced a testosterone-related variable. As a result, the logarithm of WTA/WTP ratio was modeled as the sum of the following parameters: group-level ratio μ, testosterone-related coefficient *b* proportional to the level of hormone changes *T*_*change*_, individual variation for each participant μ_*Ind*_ and item variation *g*_*goods*_
$$\log\left(\text{WTA/WTP}\right)=\mu_{0}+b*T_{change}+\mu_{ind}+g_{item}.$$

## Results

### Testosterone Concentrations

**Figure 2:**
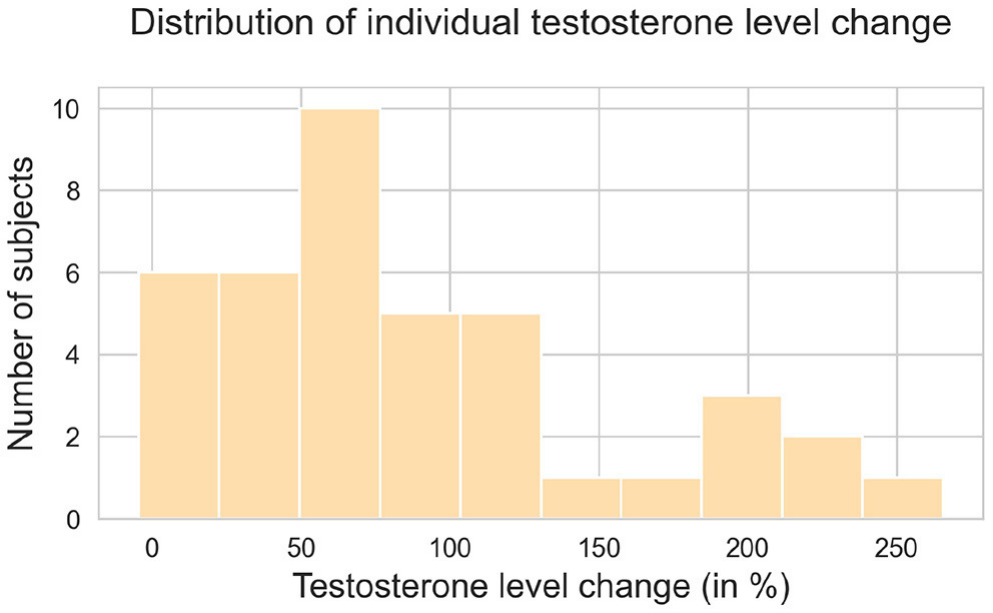
Distribution of individual testosterone level change across whole sample.

**Figure 3:**
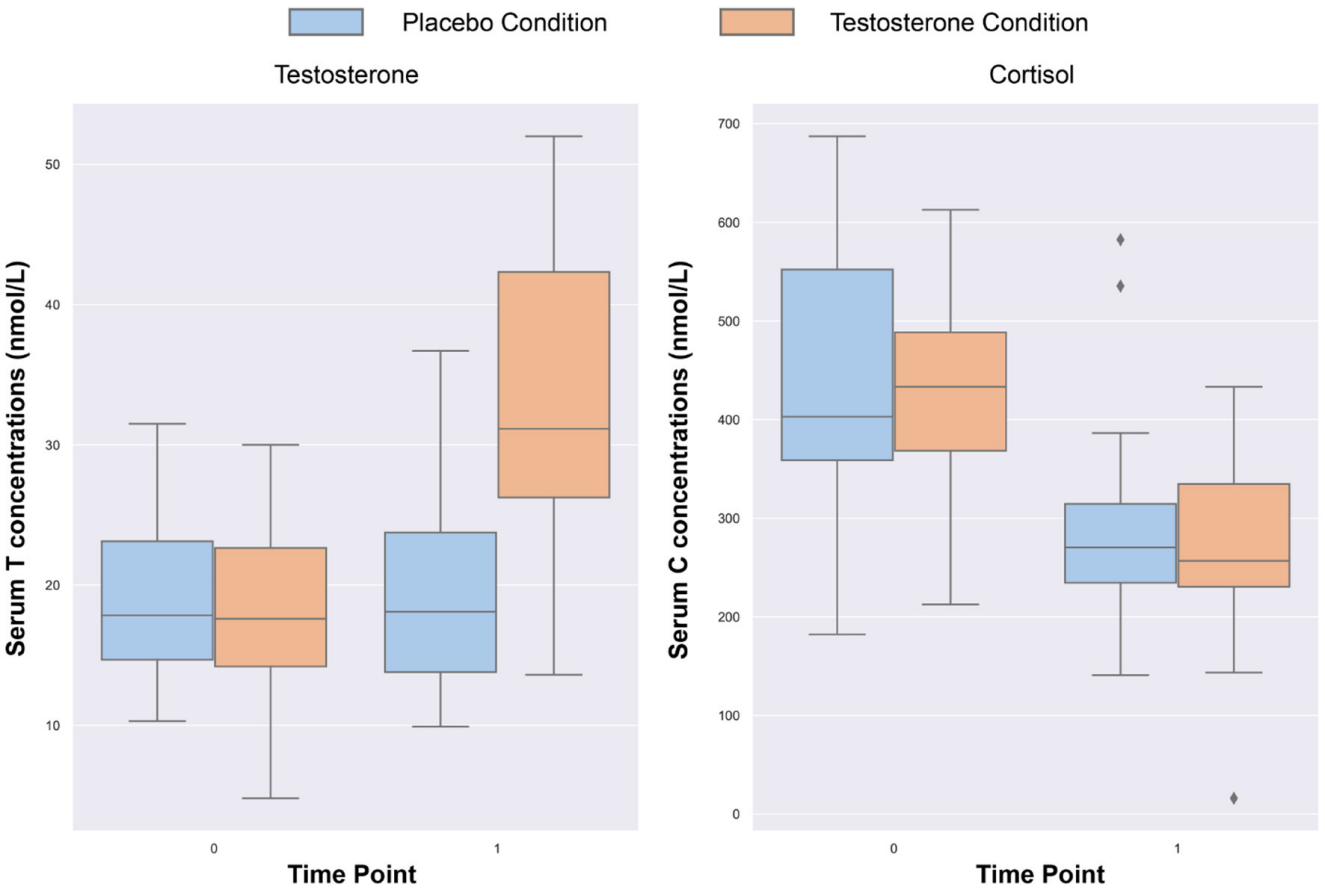
Serum level for testosterone and cortisol for 2 time points.

The repeated-measures ANOVA revealed significant main effects of treatment [$F_{\left(1,38\right)}=64.99,p<0.001,\eta^{2}=0.63$] and time [$F_{\left(1,38\right)}=84.21,p<0.001,\eta^{2}=0.69$], as well as a significant treatment-by-time interaction effect [$F_{\left(1,38\right)}=106.22,p<0.001,\eta^{2}=0.74$], indicating that testosterone levels change differently across time with transdermal testosterone relative to placebo treatment. A *post-hoc* analysis showed that serum testosterone concentration did not differ significantly between transdermal testosterone and placebo at baseline (*p* = 0.9). However, serum testosterone levels significantly increased (*p* < 0.001) in following testosterone administration at time point 1 (cca 90 min; *T*1 = 33.76 ± 11.9) compared to baseline (*T*0 = 18.53 ± 6.04). There was no difference (*p* = 0.58) in the placebo condition between baseline (*T*0 = 18.69 ± 5.44) and T1 (*T*1 = 19.0 ± 6.38), see **Figure 3**. To demonstrate individual changes of testosterone level we plotted the Figure 2. As well see before and after graphs for the placebo and testosterone averaged across all participants and per individual patient in Supplementary Figure 4.

#### Cortisol Concentrations

The repeated-measures ANOVA revealed significant main effects of time [$F_{\left(1,38\right)}=166.66,p<0.001,\eta^{2}=0.81$], indicating the known circadian fluctuation in cortisol secretion. There was no effect for treatment [$F_{\left(1,38\right)}=1,p=0.32,\eta^{2}=0.03$] nor a treatment-by-time interaction [$F_{\left(1,38\right)}=0.16,p=0.69,\eta^{2}=0.001$]. *Post-hoc* analyzes showed that serum cortisol concentration did not differ significantly between testosterone (*T*0 = 427.30 ± 97.87) and placebo treatments (*T*0 = 441.68 ± 120.25) at baseline (T0) and as well as at T1, where cortisol level in the testosterone condition was *T*1 = 273.74 ± 80.67 and in the placebo condition was *T*1 = 282.65 ± 88.12. Cortisol levels between baseline and T1 were significant for both treatments (*p* < 0.001, see Figure 3).

### Risk-Taking Under Framing

**Figure 4:**
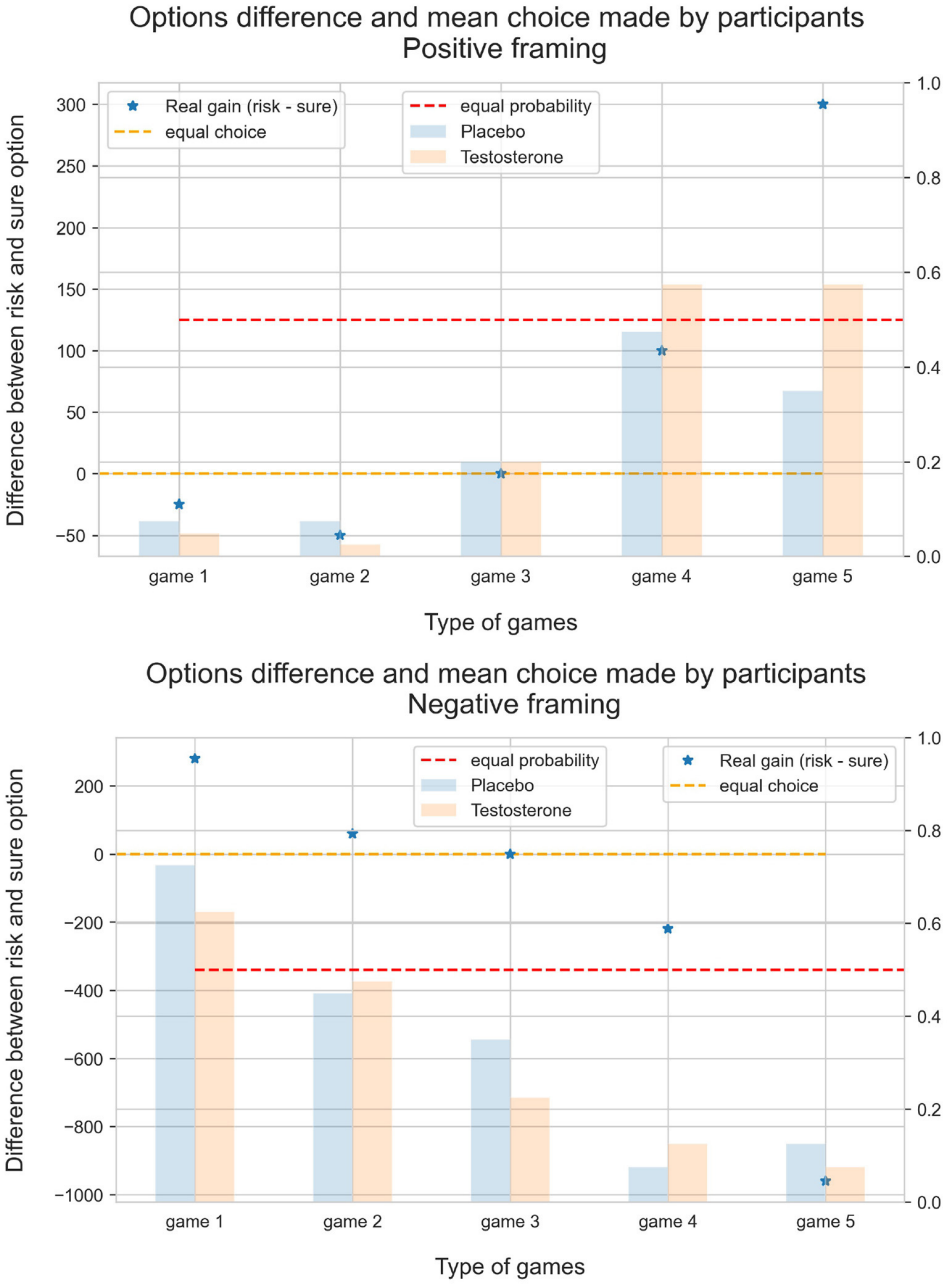
Subjective difference between risky and sure options for each game calculated as the difference between the value function for each game as defined by the CPT equation. The asterisks with different colors indicate subjective differences computed for parameters α = 0.75 and set *c* = [0.7;0.8, 0.9]. Share of risky choices made by participants plotted with the bars. From this figure we notice several properties: (1) The difference between choices is sensitive to parameter *c* (used in weighing probability function), specifically for several game options in positive framing. It even led to reversal choices in several cases; (2) Even when the risky option is much more favorable, willingness to risk stays at a level much lower than theoretically predicted; (3) In this set of games, the value range for subjective option differences lies in interval [–60,60], which corresponds to [10^−27^, 10^27^] in exponential scale. This leads to very low sensitivity coefficient and computational difficulty during estimation.

Participants chose between a risky and a sure (risk-free) option (see Table 1). Figure 4 shows the subjective difference between risky and sure options for each game calculated as the difference between the value function for each game as defined by the CPT equation. For computation, we used one parameter α = 0.75 and set *c* = [0.7, 0.8, 0.9] for close to what has been reported in several studies (for review of these studies see Fox and Poldrack (2009). Average of all choices made by participants, grouped by games, is plotted in blue for the placebo and in orange for testosterone. The CPT parameters *c*_*F*_ and α_*F*_ we re-estimated from open external dataset (Nilsson et al., 2011), with the log transform modification of game differences. The fitted replication model is available at GitHub and OSF. As a result, we used α_Positive_, α_Negative_ = 0.67, 0.96 and *c*_Positive_, *c*_Negative_ = [0.82, 0.87] as input parameters for the value function calculation.

#### Hierarchical Model Results

The hierarchical model with covariates described in the methods was estimated for recovering testosterone application effect. To verify, we estimated several additional models: pooled model (no individual differences) without testosterone parameters, pooled model with testosterone, hierarchical models with and without covariates (see in accompanying code). Adding testosterone-related parameters improved the model quality and the winning model in terms of information criterion was the hierarchical model with covariates. ROC-AUC scores scoring 1 highlight a perfect model, while random guesses yield an ROC-AUC score of 0.5. Four our baseline pooled model without testosterone-related parameters we obtained a score of 0.76 which improved when using a hierarchical model (0.94). The posterior means and standard deviations for this model parameters along with credible intervals represented in Table 2. Additionally, we provided “odds” ratio or probability of event divided by probability of no event for each parameter. It shows how the outcome changes when one of the dependent variable is changed by 1 unit. Odds equal to 1 means equal chance for both outcome.

**Table 2:** Summary of the main results of multi-level Bayesian model.

	**Mean**	**SD**	**Odds**	**hdi 3%**	**hdi 97%**
Shift (intercept) negative framing	–2.10	0.49	0.12	–3.02	–1.20
Shift (intercept) positive framing	–0.98	0.31	0.38	–1.59	–0.41
beta (negative framing)	0.63	0.14	1.87	0.37	0.89
beta (positive framing)	0.63	0.12	1.87	0.44	0.86
delta shift (negative framing)	–0.44	0.32	0.64	–1.02	0.18
delta shift (positive framing)	0.24	0.23	1.27	–0.22	0.66
delta beta (negative framing)	–0.02	0.09	0.98	–0.19	0.14
delta beta (positive framing)	0.27	0.13	1.30	0.01	0.52

*The results of estimations for risk choice modeling equation (Equation 6) Negative coefficients means that predicted probability to risk is decreasing with the covariates and vice versa. Also, 3 and 97% bounds for posterior probability for each coefficient are provided (bayesian analogous of confidence intervals). The odds show how prediction changes when features is changed by one unit. In our case, a change in relative testosterone levels by one unit in a positive framing increases the chances of choosing the game option by factor 1.27*.

**Figure 5:**
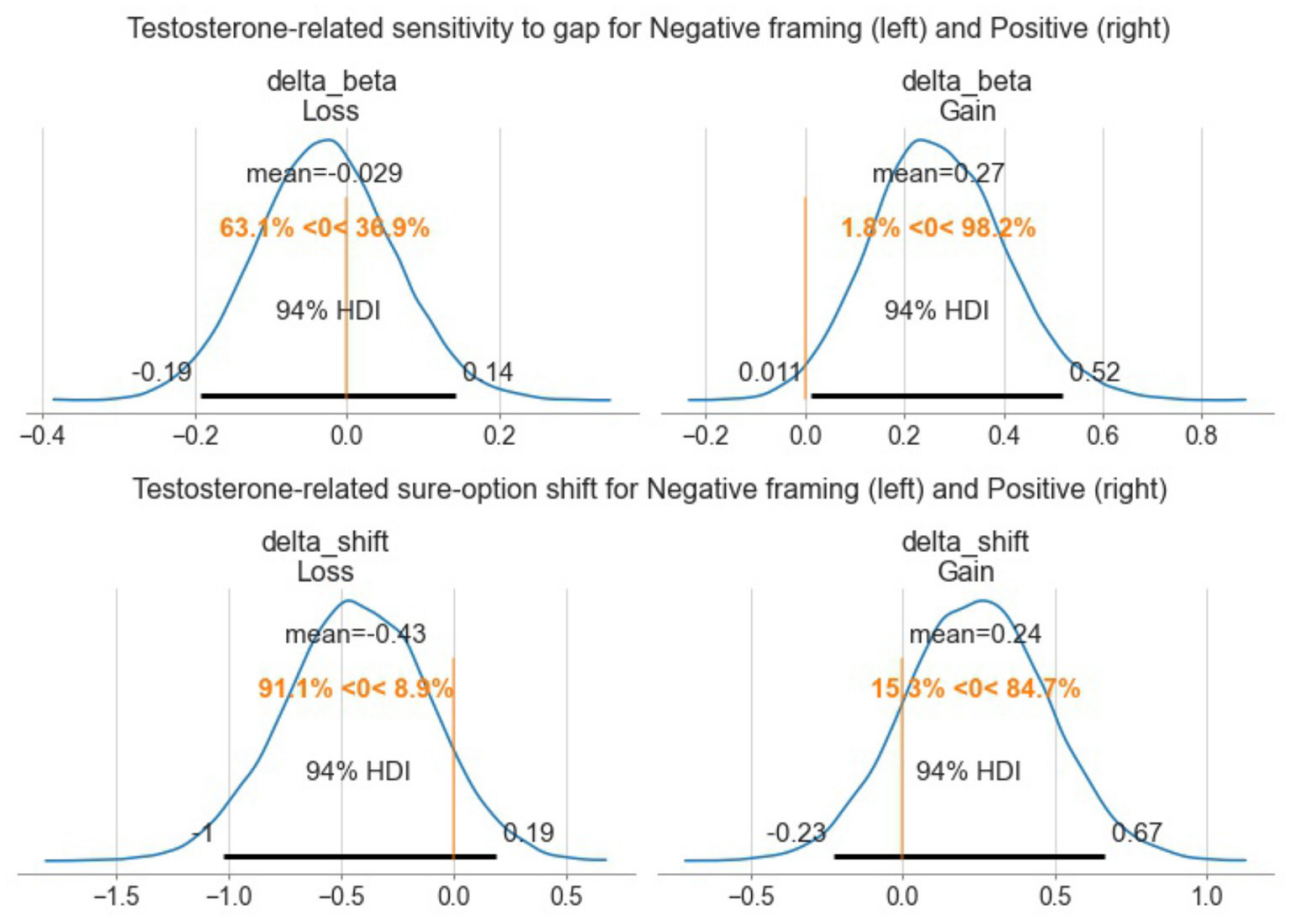
The posterior distribution for testosterone-related parameters with the zero effect indicator and 94% highest density interval (HDI).

The shift parameters responsible for preference for a risk-free option are strongly negative in both framings (94% credible interval does not contain zero value). Odds for both parameters far less than 1, what means that at in case of equal options (gap = 0) and baseline testosterone level risk-free option is strongly preferable. Second, the beta parameter or sensitivity to gain, was positive in each framing. Third, under positive framing, testosterone-related change in sensitivity was also positive (94% posterior credible interval does not contain zero; this is a reasonable amount of evidence that there is testosterone-related effects in the decision-making process). Forth, under negative framing, there was a testosterone-related shift to the risk-free (sure) option. Although the effect is small and credible interval contains zero, most of the probability mass is concentrated in the negative part. The posterior distribution for testosterone-related parameters with the zero effect indicator and 94% highest density interval is plotted in Figure 5.

#### Exploratory Analysis and Modeling of a Casual Relationship Between Changes in Serum Testosterone Level and Risky Behavior

**Figure 6:**
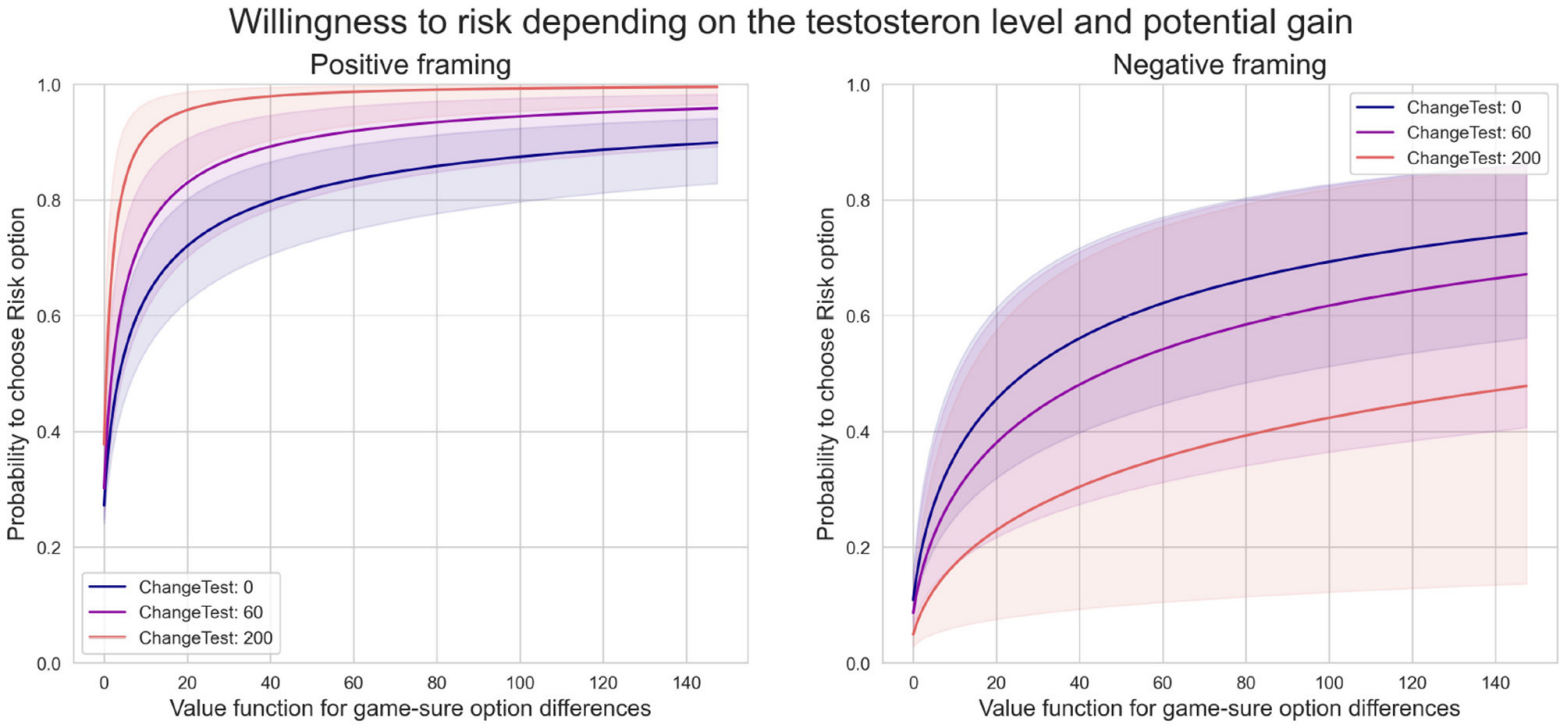
Probability of choosing risk depends on potential gain.

We plotted several counterfactual plots based on the estimated posterior distribution of parameters to explore a potential casual relationship between the change in testosterone level and risky behavior. Figure 6 highlights how the probability of choosing risk depends on fluctuations in testosterone levels. We fixed the difference in value function between risk and sure option at three levels: small (1 euro), medium (15 euro), and high (150 euro), and plotted dependencies for each level. The model shows that changes in testosterone levels shift the decision to be more risky under positive framing, particularly in small and medium levels. Under negative framing, increasing levels of testosterone have the opposite effect and decrease the probability to chose a risky option, especially for medium and high levels.

**Figure 7:**
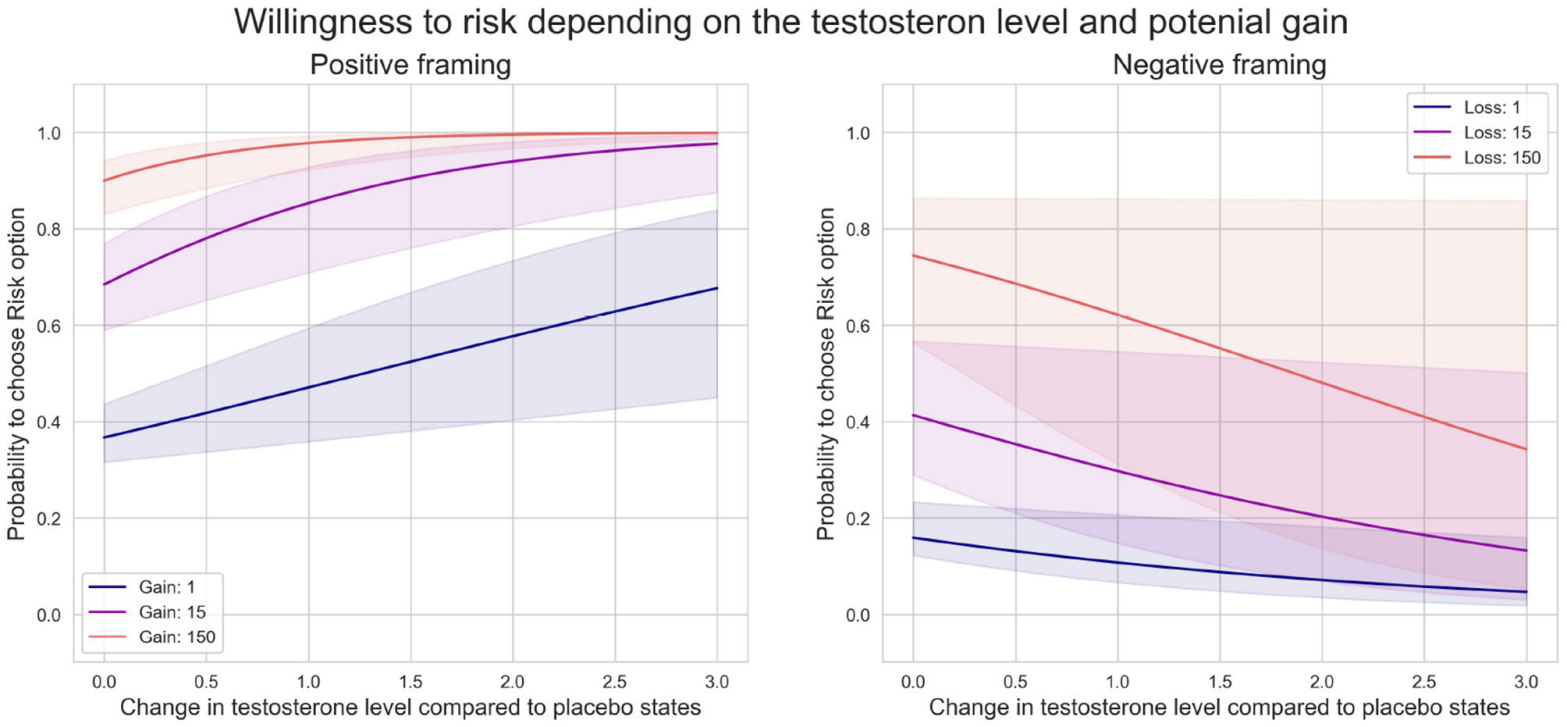
Probability of choosing risk depends on testosterone level.

In the second counterfactual simulation, we modeled the effect of three fixed testosterone levels (0, 60, and 200%) and explored risk-sensitivity to different gains. The model showed a steep shift toward riskier options under positive framing when the testosterone level increased. This further depended on the gain at stake. Under negative framing, the model also predicted an increase in risky choices when the potential loss' value increases (Figure 7). However, an increase in serum testosterone concentration diminished risk seeking.

### Endowment Effect

**Figure 8:**
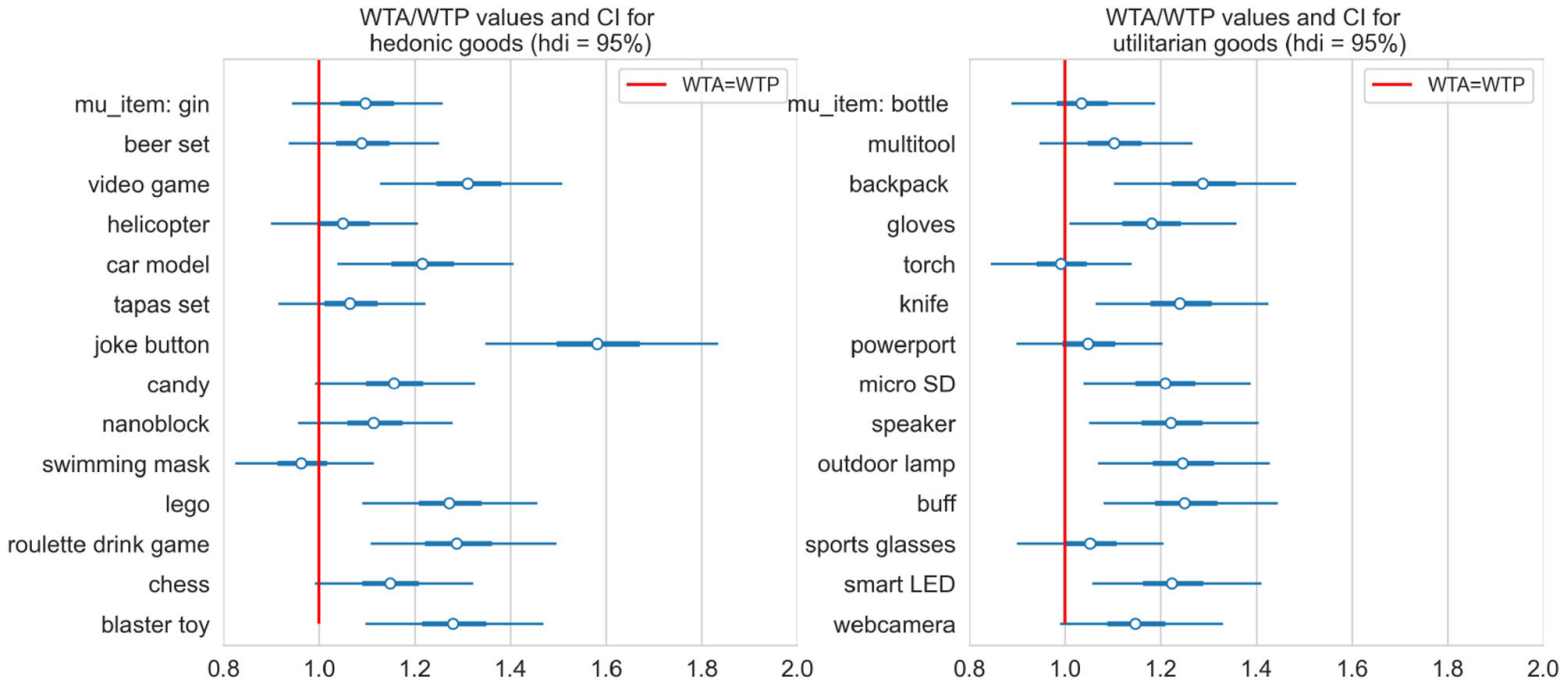
The WTA/WTP ratio for each item in the hedonic and utilitarian category in the placebo condition.

First, we examine the endowment effect regardless of the type of goods. Participants placed the WTA price higher than the WTP price for the same items even though they were aware the endowment of the product was not real. The results of one-sample *t*-test confirmed that the WTA/WTP ratio for all items was significantly higher (*p* < 0.001, Bayes Factor 1.298e+23) than for the condition without effect (WTA/WTP = 1). The WTA/WTP ratio for each item in the hedonic and utilitarian category in the placebo condition is plotted in Figure 8. These values were computed through Bayesian general linear model described in methods as WTA/WTP[*i*] = exp(μ_0_ + *g*_goods_[*i*]_).

#### Differences Between Hedonic and Utilitarian Items

**Figure 9:**
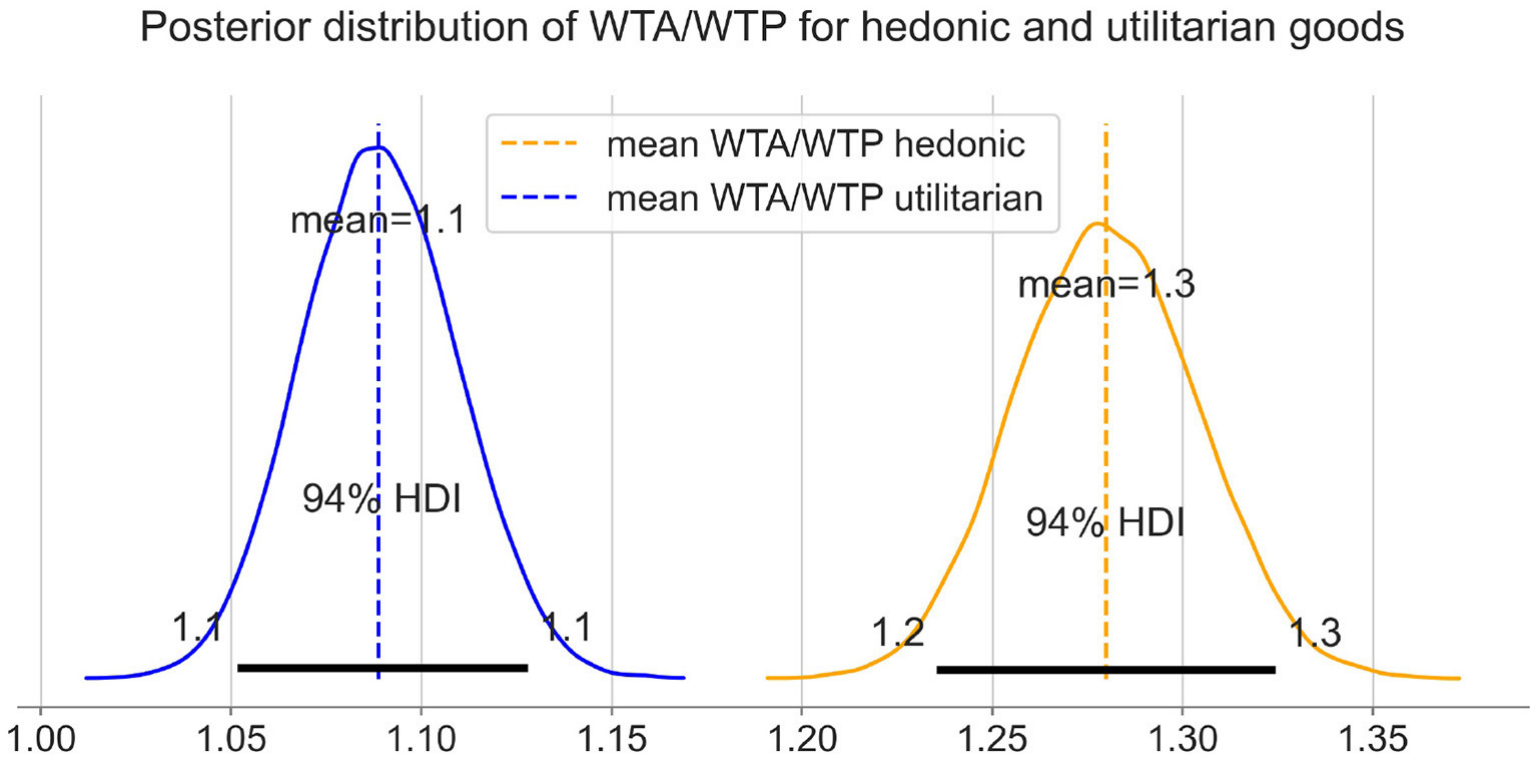
The differences between means posterior distributions for hedonic and utilitarian items.

We examined the differences between the hedonic and utilitarian categories and plotted the posterior distribution of WTA/WTP for both categories in Figure 9. The differences between means posterior distributions indicate that we have substantial evidence that the WTA/WTP ratio is higher for hedonic compared to utilitarian goods.

#### Differences Between Testosterone and Placebo

**Figure 10:**
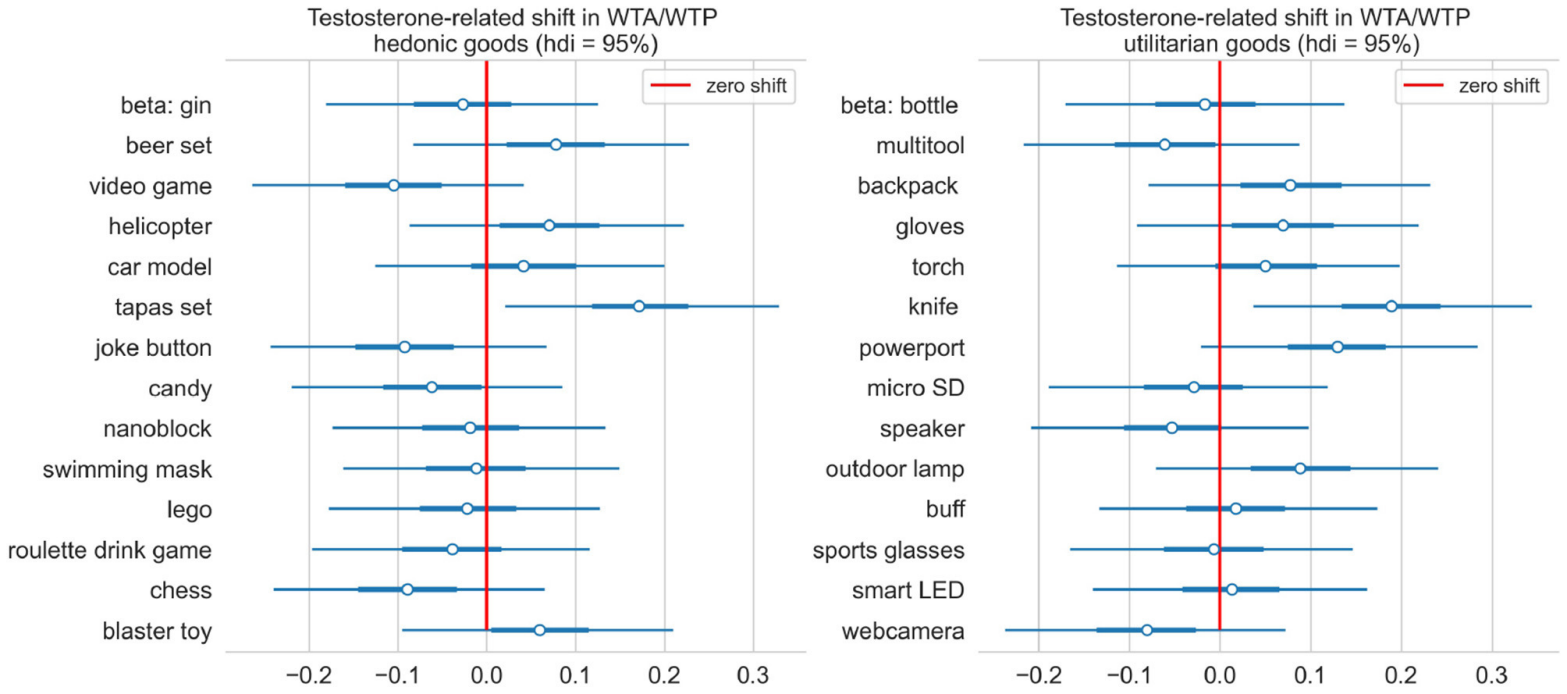
Testosterone-related single item shift int WTA/WTP, grouped by type of goods. Ninety-five percent credible interval for shift for every single item contains zero (marked with red line).

Here we examined if testosterone administration altered the endowment effect and if it affected both goods categories. After running a Markov Chain Monte Carlo sampler for the model described in the methods, the posterior distribution for each item testosterone-related shifts were approximated and high density intervals containing 95% of probability were plotted in Figure 10. While we observed slight testosterone-related shifts for some items in both categories, we did not find significant differences between the two treatment groups. Overall, we observed an endowment effect for all items regardless of their category. The endowment effect, however, was stronger for hedonic compared to utilitarian items.

#### Effect Size

Testosterone related treatment in each framing option consists of two coefficients: a basic preference for sure options and the sensitivity to gain or loss. The effect size for testosterone treatment, computed as a coefficient value relative to its standard deviation, in the positive framing is 1.04 (large) and 2.07 (very large) for the basic preference for sure options and the sensitivity to gain size. In the negative framing, both coefficients have negative signs with the effect sizes equal to –1.34 (large) and –0.2 (ignored) for basic preference for sure loss and sensitivity to loss size.

## Discussion

Using frequentist and Bayesian modeling approaches, this study investigated the effects of testosterone administration on risk-taking decision-making under positive and negative framing and the role of testosterone in the endowment effect. Treatment manipulation was successful and we observed a significant increase in serum testosterone concentrations 1.5 h after transdermal application. Compared to placebo, testosterone administration increased risk-taking under the positive framing and decreased under the negative framing. Our model showed that decision-making is jointly influenced by testosterone and the trade-off between gains and losses. Moreover, we observed the expected endowment effect which was more pronounced for hedonic than for utilitarian goods. Unlike our predictions, however, testosterone did not alter the endowment effect.

There is ongoing debate as to whether testosterone increases risky behavior and choice. Our aim was to examine whether testosterone administration affects risky behavior in a series of games under positive and negative framing (gain and loss) that were associated with different probabilities. We expected participants to show increased risk-seeking behaviors regardless of framing type but we only observed this pattern in the positive framing condition.

Several studies showed a causal or correlational effect of endogenous and exogenous testosterone on increased risk-taking behaviors. For instance, high endogenous testosterone levels correlated with increased risk-taking in both genders and with the willingness to take higher financial risks (Garbarino et al., 2011; Chicaiza-Becerra and Garcia-Molina, 2017). Similarly, baseline endogenous testosterone levels were associated with increased risk-taking (Stanton et al., 2011a) showing that both women and men with higher testosterone levels acted riskier than the groups having lower testosterone levels. Similar results were observed in studies that altered testosterone levels pharmacologically. For instance, exogenous testosterone increased risk-taking in the Iowa Gambling Task (van Honk et al., 2004) by preferentially modulating the punishment and reward sensitivities.

While several studies showed a positive association between basal testosterone or pharmacologically elevated testosterone and risk-taking (van Honk et al., 2004; White et al., 2006; Goudriaan et al., 2010; Apicella et al., 2014; Evans and Hampson, 2014), there is also evidence of null results (Zethraeus et al., 2009; Boksem et al., 2013; Ortner et al., 2013; Van der Loos et al., 2013; Derntl et al., 2014) or even negative correlations (Stanton et al., 2011b; van Anders et al., 2012). However, direct causal comparisons between various studies investigating risk-taking and testosterone are limited by methodological constraints that may hamper interpretations. For instance, the doses used to pharmacologically elevate testosterone levels are heterogeneous within and between sexes (i.e., in men, exogenous applications vary from 50 to 150 mg transdermal doses vs. the standardized 0.5 mg sublingual administration in women). Likewise, endogenous measures of testosterone levels are computed using 2D:4D ratios, saliva, blood, or hair sampling that all have inherent limitations.

It should be considered that other factors may play a role as well. The study using the Iowa Gambling Task showed a joint role of cortisol and testosterone in decision-making in the Singh (2021). Specifically, cortisol impaired decision-making in trials with high uncertainty, while testosterone improved decision-making in high risk trials. Several other studies examined the effect of testosterone using the Balloon Analog Risk Task (BART). Goudriaan et al. (2010) examined the effects of testosterone after treating participants with an aromatase inhibitor (letrozole 2.5 mg) for a week and found increased risk-taking in the high testosterone group in the BART but not in the Iowa Gambling Task (Goudriaan et al., 2010). Moreover, Wagels and colleagues (2017) found an interaction between MAOA polymorphism and testosterone and showed that MAOA-S carriers played riskier than the MAOA-L carriers (Wagels et al., 2017).

Only a handful of studies used a similar approach to ours in testing risk-taking behaviors using binary decision games. Stanton et al. (2011b), for instance, measured testosterone levels in participants who performed a risk preference, loss aversion, and ambiguity task and found a U-shaped effect for risk and ambiguity. In other words, participants with low and high testosterone levels were risk- and ambiguity-neutral, whereas individuals with intermediate testosterone levels were risk- and ambiguity-averse. These findings contradict our results where we observed increased risk-taking behavior under positive framing. Our model showed that the shift to risky behavior depends on the individual level of testosterone fluctuation, the gain at stake, and the probability of obtaining this gain. We observed a reverse behavioral pattern under negative framing. Contrary to our predictions, testosterone administration tuned participants' behavior to be more risk-averse. Regardless of treatment (placebo or testosterone), however, participants were more risk-taking when the prospected financial gains were higher. As such, we suggest that participants follow an individual outcome optimization strategy and reduce potential loss when inevitable while increasing potential gains when there is a chance for it. This optimization strategy, however, is less susceptible to hormonal influence and more in line with contextual factors. Nadler et al. (2021) presented participants with two gambles under positive framing. Participants could select either a sure option to get $1 or gamble with obtaining $3 with a varying probability between 0.1 and 1 or getting nothing (Nadler et al., 2021). They excluded from analysis participants who showed intransitive preferences and classified the remaining participants into three risk-taking levels: risk-averse, slightly risk-averse, and risk-loving. Their analyzes revealed similar risk-taking profiles and no treatment effects. Another recent study by Stanton et al. (2021) similarly found no consistent relationship between testosterone and economic decision-making. Since testosterone does not work in isolation, it is likely that potential contextual moderators may alter the circumstances under which testosterone affects economic decision-making.

In the endowment task participants state their monetary preference for buying and selling various items falling into hedonic or utilitarian categories. Results showed that the WTA/WTP ratio was significantly higher and thus we observed the predicted endowment effect. The finding aligns with previous literature (Thaler, 1980; Knetsch, 1989; Kahneman et al., 1991; Van Dijk and Van Knippenberg, 1998; Carmon and Ariely, 2000; Votinov et al., 2013; Gächter et al., 2021) that demonstrated an endowment effect across various types of goods (real owned and imaginary) as well for different types of transactions (goods-to-money, goods-to-goods). Moreover, as predicted, we found a larger endowment effect for hedonic compared to utilitarian items. Previous findings argued that owning hedonic items might facilitate the development of a symbolic relationship to the items compared to items serving a clear purpose (i.e., utilitarian; Belk, 1988). This emotional attachment toward an item can be associated with more positive emotions and increased aesthetic value which might resemble the feelings hedonic items yield (Ariely et al., 2005).

Testosterone administration did not alter the endowment effect in general nor did alter the preference for goods. Investigating items individually, we found an increase in WTA/WTP ratio only for a few of the items in both groups. We believe this effect could be item specific and bound to the subjective value individuals ascribed to them or, alternatively, could be random noise. Previous studies found that the effect of testosterone on consumer behavior was associated with status or owning luxury goods that served to attract mates, intimidate potential rivals (Nepomuceno et al., 2016a,b) or change product preferences (Aspara and Van Den Bergh, 2014). However, Wu and colleagues (2017) tested the association between competition, testosterone fluctuation, and conspicuous consumption and found no evidence for a hormonal regulation of these effects (Wu et al., 2017). Nevertheless, winners in the competition showed increased self-reported WTP and an implicit bias toward high-status products. These studies, however, measure testosterone levels predominantly using prenatal markers (i.e., 2D:4D ratio) that have limited ecological validity (Berenbaum et al., 2009; Nadler et al., 2021). Thus, one cannot rule out the situational effects of decision-making that may be additive to increased endogenous testosterone concentrations. Future research should address the association between the type of consumer goods and test real items that participants own while controlling for their emotional attachment to a given item (e.g., childhood toy or present from a beloved one).

## Limitations

Our results should be interpreted in the light of several limitations. Only young, healthy men took part in this study. Moreover, due to inherently different hormonal profiles, the results cannot be generalized to women. Nevertheless, prior animal model and human research suggests that acute testosterone effects are expected to manifest predominantly in men due to the distinct hormonal profiles. Last, we did not have an endogenous measure of testosterone concentration prior to the testing sessions (2D:4D measurement) to correlate with baseline intake testosterone levels. There is evidence that prenatal testosterone plays a strong role in the activational effects of testosterone for higher-order social cognition (Terburg et al., 2016). Nevertheless, 2D:4D ratio results regarding testosterone effects are rather heterogenous and susceptible to measurement errors/bias. Future studies should consider other factors which may influence testosterone concentrations, such as levels of enzyme aromatase, sex hormone binding globulin (SHBG) and expression of androgen receptors (AR).

## Conclusions

This study showed the effect of testosterone administration on risk-taking behavior in young healthy men. Testosterone increased risk-taking behaviors under positive framing and risk aversion under negative framing. However, the sensitivity to gain remained positive in each framing. We found evidence for the endowment effect which was increased for hedonic com- pared to utilitarian items. This effect was independent of testosterone. Our results underline the modulatory role of testosterone on risk-taking within different framing. The models we tested can be used as predictive models for risk-taking behavior and may be applied in larger and more diverse samples. Further studies should examine the gender-specific aspects of risk preference and the link to the modulatory effects of testosterone among males and females. Additionally, the effect of different age groups on risk preference warrants further research considering that the testosterone concentrations vary over the lifespan, specifically in men. This could further be extended beyond financial risk-taking to other risky behaviors (i.e., exploration, promiscuity, etc.).

## Data Availability Statement

The datasets presented in this study can be found in online repositories. The names of the repository/repositories and accession number(s) can be found in the article/Supplementary Material.

## Ethics Statement

The studies involving human participants were reviewed and approved by Ethics Committee of the Medical Faculty, RWTH Uniklinik. The patients/participants provided their written informed consent to participate in this study.

## Author Contributions

MV and AP designed the experiment. AP performed the experiment. IK and MV analyzed the data. MV, IK, and AP wrote the manuscript. MV, IK, UH, KK, and AP revised the manuscript and provided critical feedbacks. All authors contributed to the article and approved the submitted version.

## Funding

AP was supported by a scholarship from the International Research Training Group. The Neuroscience of Modulating Aggression and Impulsivity in Psychopathology (IRTG-2150) of the German Research Foundation (DF'Projektnummer 269953372/GRK2150).

## Conflict of Interest

The authors declare that the research was conducted in the absence of any commercial or financial relationships that could be construed as a potential conflict of interest.

## Publisher's Note

All claims expressed in this article are solely those of the authors and do not necessarily represent those of their affiliated organizations, or those of the publisher, the editors and the reviewers. Any product that may be evaluated in this article, or claim that may be made by its manufacturer, is not guaranteed or endorsed by the publisher.
