# Supplementary material for: A Bayesian Modeling Approach to Examine the Role of Testosterone Administration on the Endowment Effect and Risk-Taking
Source: Front Neurosci. 2022 Jul 20;16:858168. doi: 10.3389/fnins.2022.858168 (PMC9347223; doi:10.3389/fnins.2022.858168)
Supplement: Supplementary file 1 [file Data_Sheet_1.pdf]

Appendix: A Bayesian Modelling Approach to Examine the Role of Testosterone Administration on the Endowment Effect and Risk-Taking

Mikhail Votinov [1,2*✉], Irina Knyazeva [3,4*], Ute Habel [1,2], Kerstin Konrad [5], and Andrei A Puiu [2✉]

[1]Institute of Neuroscience and Medicine: JARA-Institute Brain Structure Function Relationship (INM 10), Research Center Jülich, Jülich, Germany
[2]Department of Psychiatry, Psychotherapy and Psychosomatics, Faculty of Medicine, RWTH Aachen University, Aachen, Germany
[3]N.P. Bechtereva Institute of Human Brain, Russian Academy of Science, Saint-Petersburg, Russia
[4]Saint-Petersburg State University, St.Petersburg, Russia
[5]Department of Child and Adolescent Psychiatry, Psychosomatics and Psychotherapy, Faculty of Medicine, RWTH Aachen University, Aachen, Germany
[*]authors contributed equally

1 Bayesian models estimation details

Below we provided specification for Bayesian model used in paper. Despite the power of hierarchical models, there are a lot of subtle problems appearing during the estimation of these models, and to overcome convergence problems we used a so-called "reparameterization trick", specifically non-centered parametrization McElreath (2018). The only difference is in the way of model formulation, in case of Normal distribution with parameters μ and σ we can sample directly from Normal(μ, σ) or from $\mu + \sigma * (\text{Normal}(0,1))$. The outcome is absolutely identical, but sampling procedure differs. So, we used non-centered priors in all the models. Also, for all the models posterior distributions were approximated by a total of 24,000 MCMC samples obtained from four chains, after a burn-in of 1000 samples. Convergence of the MCMC chains was confirmed by visual inspection and by computing relevant R hat statistics.

Replication study

Open dataset explored in Nilsson et al. (2011) includes 30 participants and 180 games, each game is a chance prospect with two non-zero outcomes $x, p; y, (1-p)$, data available at `https://osf.io/sbxm2/`. In those games there were 60 games with pure gain prospects, 60 with pure loss and 60 with mixed prospects. Apart from our study both options A, B are chance

prospects, and there is no reason to prefer one of them by default, so we excluded intercept parameter . We replicated the hierarchical model suggested in the original paper, but only for pure gain and pure loss (positive and negative framing) games, because we did not have mixed games in our study. We also estimated the model with log transform modification.

From prospect theory (see section Prospect Theory) in case of two games with different non-zero probabilities:

$$V(A) = \sum v(x_i) * \omega(p_i)$$

where $V(A)$ - value function, $\omega(p_i)$ - decision weights for for gains and losses

$$v(x_i) = \begin{cases} x^{\alpha_{cond}}, & \text{if}(x) > 0 \\ -\lambda(-x)^{\alpha_{cond}}, & \text{else} \end{cases}$$

$$\omega(p) = \frac{p^{c_{cond}}}{(p^{c_{cond}} + (1-p)^{c_{cond}})^1/c_{cond}}$$

where $cond = +,$ if positive $, -$ else is a game type

Choice function, probability of choosing option B over A:

$$\theta = \frac{1}{1 + e^{-\phi(V(B) - V(A))}}$$

We slightly modify the equations for computational purpose. We considered only pure loss and pure win games, so we can use different ϕ for each treatment instead of λ:

$$V(B) - V(A) = \lambda * (\ldots)$$

so we can use ϕ_{cond}, where $\phi_- = \lambda * \phi_+$

Model specification

* Likelihood:

- $y_i \sim$ Bernoulli(θ_i) (binary outcome)
- Choice probability for game with two outcomes (x,y), with the second outcome

probability equal to q modeled with logistic regression.

$$\begin{cases} \text{logit}(\theta_i) = \phi_{ind} * \text{gap}(A, B, \alpha_{ind}, c_{ind}) \\ \text{gap}(A, B) = \text{Val}(B, \alpha_{ind}, c_{ind}) - \text{Val}(A, \alpha_{ind}, c_{ind}), \text{original model} \\ \text{gap}(A, B) = \text{sign}(\text{gap}(A, B)) * \log(1 + \text{abs}(\text{gap}(A, B))), \text{modification used in this paper} \\ \text{Val(Game} = x, 1-q, y, q) = (1 - w_q(c_{cond}))v(x, \alpha_{cond}) + w_q(c_{cond})v(y, \alpha_{cond}) \end{cases}$$

* Priors. All the model parameters have constraint boundaries, α and c can be in the range [0,1] ϕ could take only positive values. So, we couldn't take the normal distribution for priors. However, on the probit scale the parameters cover the entire real line, so additional normally distributed variables were introduces and transformed after that with probit scale to [0-1] range and exponentially to positive range. Individual differences introduces with hierarchical model, but unlike original paper we used non-centered parametrisation. Precisely, the individual parameters come from independent group-level normal distributions

  - Priors for α parameter which modulate the curvature of the subjective value functions, group level and individual:

$$\begin{cases} \alpha N_{cond} \sim \text{Normal}(0, 5, \text{size} = 2), \\ \alpha_{cond} \sim \text{probit}(\alpha N_{cond}) \end{cases}$$

  - Priors for c specifies the transformation of the weighting function, group level and individual:

$$\begin{cases} cN_{cond} \sim \text{Normal}(0, 5, \text{size} = 2) \\ c_{cond} \sim \text{probit}(cN_{cond}) \end{cases}$$

  - Priors for sensitivity parameter ϕ:

$$\begin{cases} \phi N_{cond} \sim \text{Normal}(0, 5, \text{size} = 2) \\ \phi_{cond} \sim \exp(\phi N_{cond}) \end{cases}$$

- Non-centered priors for individual differences in parameters:

$$\begin{cases} \alpha_{ind} = \text{probit}(\alpha N_{cond} + z_{\alpha_{ind}} * \sigma_{\alpha_{ind}}) \\ c_{ind} = \text{probit}(c N_{cond} + z_{c_{ind}} * \sigma_{c_{ind}}) \\ \phi_{ind} = \exp(\phi N_{cond} + z_{\phi_{ind}} * \sigma_{\phi_{ind}}) \end{cases}$$

Risk-taking model

The measured data from the experiment is presented as binary choice outcomes, so the data generation process is a binomial distribution.

* Likelihood:

- $y_i \sim$ Bernoulli(θ_i), with θ probability of taking risk in each game
- The probability θ is modelled with logistic regression

$$\text{logit}(\theta) = \text{Intercept} + \text{Beta} * \text{gap}(A, B, \alpha, c)$$

- For parameters A and B, there is a variety of options for modelling. Since both slope and intercept are considered a function of framing (F) and testosterone application, we added it as a linear term multiplied by the rate of change in testosterone level relative to placebo denoted as Tchange below.

$$\begin{cases} \text{Intercept} = a_F + \delta\_a_F * \text{Tchange} \\ \text{Beta} = b_F + \delta\_b_F * \text{Tchange} \end{cases}$$

* Priors specification In our model, we suppose that there is a framing-dependent group-level a_F sampled from a normal distribution. Individual parameters a_{ind} sampled from a normal distribution with group-level mean and standard deviation. The same stands true for B, which is the sensitivity to payout difference. However, it is unreasonable to suggest negative sensitivity to the gap, so we also choose normal prior but with 0.5 mean

and standard deviation equal to 0.5. For testosterone-related terms δ_a_F and δ_b_F we did not use a hierarchical approach, because individual differences mostly were taken into account through independent variable Tchange - change in testosterone level. For them, it is also natural to choose normal priors with zero mean and unit variance. As a result, we have four risk-taking related group-level parameters, and four testosterone risk-taking related. Since it is natural for an individual to suggest interdependence from all parameters, we suppose that a risk-seeking subject will demonstrate it in both framings or there will at least be some correlation, both for intercept and slope parameters. Also, we take into account the covariation between intercepts and slopes in each framing. Formally,

$$A \sim \text{MvNormal}([a_F + \delta\_a_F, b_F + \delta\_b_F], \Sigma)$$

Figure with the model description 1:

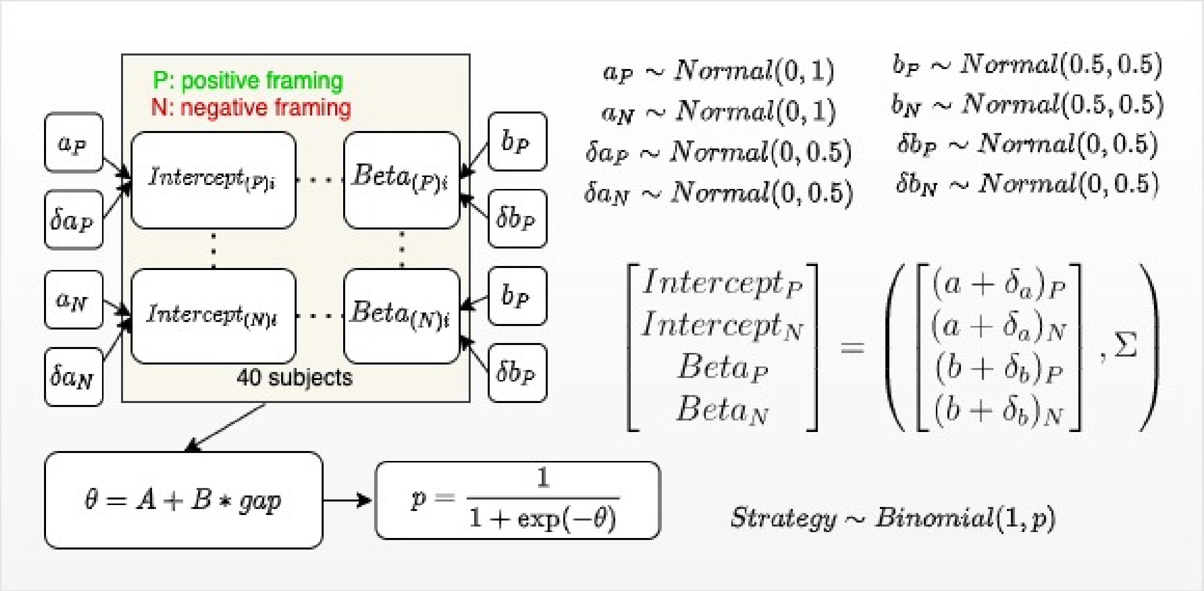

Figure 1: Prior specification for risk-taking model

Endowment effect model specification

The WTA/WTP ratio measures endowment effect. In this paradigm, willingness to pay (WTP) to acquire an object is typically lower than the least amount they are willing to accept (WTA) to give up that same object when they own it—even when there is no cause for attachment. The

hypothesis to test:

1. WTA/WTP > 1 There is endowment effect for both type of goods (utilitarian and hedonic)

2. WTA/WTP$_h >$ WTA/WTP$_u$ Endowment effect for hedonic goods bigger then for utilitarian

3. WTA/WTP$_T >$ WTA/WTP$_P$ Testosterone administration will increase endowment effect, especially for hedonic goods.

For these hypothesis testing we estimated two models. The first one for endowment effect existence for two type of goods regardless individual and item differences and without testosterone-related parameters included and the second one with individual and item differences and with testosterone-related shift parameter.

Model 1

Bayesian model: WTA/WTP ratio came from lognormal distribution with unknown mean and variance. Model specification:

* **Likelihood**: WTA/WTP $\sim \text{LogNormal}(\mu_{\text{Type}}, \sigma_{\text{Type}})$

* **Priors**: informative prior for checking H0 hypothesis: mean = 0, what corresponds 1 (equal WTA and WTP) in real scale, because in logarithmic because $\exp(0) = 1$. Formally:

  - $\mu \sim \text{Normal}(\text{mean} = 0, \sigma = 1, \text{size} = 2)$, mean prior
  - $\sigma \sim \text{Exponential}(1, \text{size} = 2)$, uniformative prior for sigma

Lognormal distribution for WTA/WTP ratio was chosen because ratio is strongly positive and skewed as evidenced by empirical distributions as could be seen at the Fig. 2

For assessing the plausibility of the model we draw posterior predictive distribution for the WTA/WTP ratio based on the fitted Bayesian model. We can see from the Fig. 3 that observed data set is consistent with the model.

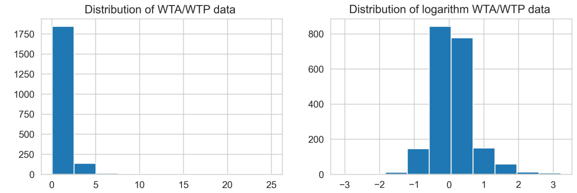

Figure 2: Histogram for raw WTA/WTP data and their logarithm

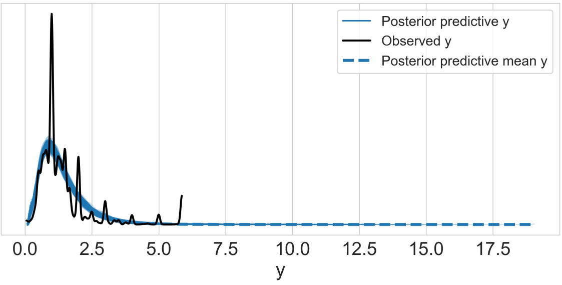

Figure 3: Posterior predictive distribution for Model 1

Model 2

In this model we used the same we used hierarchical approach and added item differences and individual differences at the second level and item differences and with testosterone-related shift parameter Tchange computed as relative difference in testosterone level for placebo and hormone condition for each participant.

* **Likelihood**: $\log(\text{WTA/WTP}_{[i,j]}) = \mu_0 + \beta * Tchange + \mu_{\text{ind}[j]} + g_{\text{g}[i]}$

* **Priors**:

  - $\mu_0 \sim \text{Normal}(0, 0.5)$ (group mean)
  - $\sigma \sim \text{Exponential}(1)$ (model error)
  - $\sigma_g = \text{Exponential}(2)$ (between item deviation)
  - $\sigma_{ind} = \text{Exponential}(2)$ (between subject deviation)
  - $\beta \sim \text{Normal}(0, 0.5, size = \text{Nitems})$ (sensitivity to testosterone level change, different for each item)
  - $z_{\text{g}} \sim \text{Normal}(0, 1, size = \text{Number of items})$, $g_{\text{g}} = z_{\text{g}} * \sigma_{\text{g}}$ (item mean shift)
  - $z_{\text{ind}} \sim \text{Normal}(0, 1, size = \text{Number of subjects})$, $\mu_{\text{ind}} = z_{\text{ind}} * \sigma_{\text{ind}}$ (subject mean shift)

Bibliography

R. McElreath. *Statistical rethinking: A Bayesian course with examples in R and Stan*. Chapman and Hall/CRC, 2018.

H. Nilsson, J. Rieskamp, and E.-J. Wagenmakers. Hierarchical bayesian parameter estimation for cumulative prospect theory. *Journal of Mathematical Psychology*, 55(1):84–93, 2011.

Results part

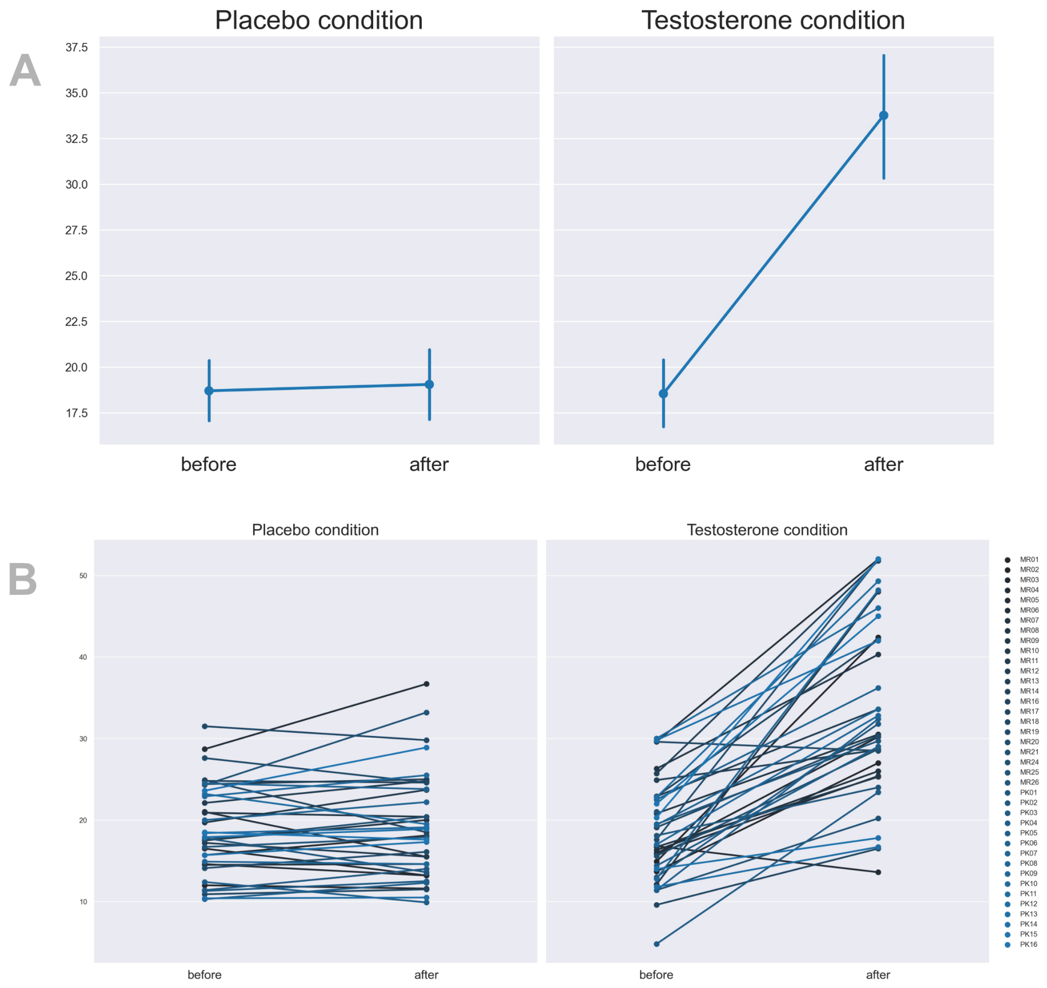

Figure 4: Testosterone level changes a) all subjects for Placebo and Testosterone condition b) for each subject for both conditions.
